# A comparative trial of blood pressure monitoring in a self-care kiosk, in office, and with ambulatory blood pressure monitoring

**DOI:** 10.1186/s12872-023-03701-1

**Published:** 2024-01-03

**Authors:** Gunnar Nilsson, Anna Lindam

**Affiliations:** 1 Department of Public Health and Clinical Medicine, Umeå University, 905 81 Umeå, Sweden; 2 Department of Public Health and Clinical Medicine, Unit of Research, Education, and Development, Östersund Hospital, Umeå University, Umeå, Sweden

**Keywords:** Ambulatory blood pressure, Self-blood pressure monitoring, Clinical trial

## Abstract

**Background:**

Automated measurement of blood pressure (BP) in designated BP kiosks have in recent years been introduced in primary care. If kiosk blood pressure (BP) monitoring provides results equivalent to in-office BP or daytime ambulatory BP monitoring (ABPM), follow-up of adult patients could be managed primarily by self-checks. We therefore designed a comparative trial and evaluated the diagnostic performance of kiosk- and office-based BP (nurse- versus physician-measured) compared with daytime ABPM.

**Methods:**

A trial of automated BP monitoring in three settings: a designated BP kiosk, by nurses and physicians in clinic, and by ABPM. The primary outcome was systolic and diastolic BP, with respective diagnostic thresholds of ≥135 mmHg and/or ≥ 85 mmHg for daytime ABPM and kiosk BP and ≥ 140 mmHg and/or ≥ 90 mmHg for office BP (nurse- and physician-measured).

**Results:**

Compared with daytime ABPM, mean systolic kiosk BP was higher by 6.2 mmHg (95% confidence interval [CI] 3.8–8.6) and diastolic by 7.9 mmHg (95% CI 6.2–9.6; *p* < 0.001). Mean systolic BP taken by nurses was similar to daytime ABPM values (+ 2.0 mmHg; 95% CI − 0.2–4.2; *p* = 0.071), but nurse-measured diastolic values were higher, by 7.2 mmHg (95% CI 5.9–9.6; *p* < 0.001). Mean systolic and diastolic physician-measured BPs were higher compared with daytime ABPM (systolic, by 7.6 mmHg [95% CI 4.5–10.2] and diastolic by 5.8 mmHg [95% CI 4.1–7.6]; *p* < 0.001). Receiver operating characteristic curves of BP monitoring across pairs of systolic/diastolic cut-off levels among the three settings, with daytime ABPM as reference, demonstrated overall similar diagnostic performance between kiosk and nurse-measured values and over the curve performance for physician-measured BP. Accuracy with nurse-measured BP was 69.2% (95% CI 60.0–77.4%), compared with 65.8% (95% CI 56.5–74.3%) for kiosk BP.

**Conclusions:**

In this study kiosk BP monitoring was not comparable to daytime ABPM but could be an alternative to in-office BP monitoring by trained nurses. The diagnostic performance of kiosk and nurse-measured BP monitoring was similar and better than that of physician-measured BP.

**Trial registration:**

ClinicalTrials.gov (NCT04488289) 27/07/2020.

**Supplementary Information:**

The online version contains supplementary material available at 10.1186/s12872-023-03701-1.

## Background

Monitoring of hypertension is a core task in primary practice. The worldwide hypertension prevalence, defined as systolic blood pressure (BP) ≥140 mmHg or diastolic BP ≥90 mmHg or use of anti-hypertensive medication, is estimated to be about 30% in adults [1] and remains a predominant cardiovascular risk factor [2, 3]. Care of hypertensive patients in accordance with current guidelines [4] imposes challenges in treatment, monitoring, and comorbidity-management strategies [5–10]. Complementary techniques for BP monitoring, such as ambulatory BP monitoring (ABPM) and automated measurement in designated BP kiosks or at home, have gradually been introduced [11–17]. The rationale has been to increase patient engagement and reduce a potential white-coat effect on BP monitoring. According to previous findings, there is an expected difference in systolic BP of 5–15 mmHg between manual and automated office measurement, with automated BP being lower [18]. How mean values recorded in a BP kiosk correspond to daytime ABPM or to office-measured BP has not been sufficiently evaluated in practice. Currently, both ABPM and kiosk BP monitoring are commonly offered to patients in Swedish primary care. However, standardized home monitoring of hypertension had not been implemented in our setting by the time of the current study, despite recommendations [4, 16].

ABPM has the disadvantage of being dependent on special equipment, logistic conditions, and patient acceptability [19]. If kiosk BP monitoring provides results that are equivalent to daytime ABPM values, follow-up of patients could be managed primarily by self-checks, to support the treatment decisions by GPs and other physicians.

We therefore examined data for primary care patients treated or investigated for hypertension in parallel with three different techniques: automated BP measurement in a designated BP kiosk, automated measurement in the clinic by nurses and physicians, and ABPM for 24 hours.

The main study aim was to assess BP monitoring in a kiosk for self-determined BP measurement and compare in-office BP monitoring between nurses and physicians and with daytime ABPM as reference. A secondary aim was to evaluate the diagnostic performance of kiosk-based BP and office BP compared with ABPM.

## Methods

### Setting and population

Study participants, all age 20 years or older, were recruited at Föllinge Health Centre (population 1500), located in a rural area in Region Jämtland Härjedalen, northern part of Sweden. Eligible study participants were primary care patients treated for essential hypertension (International Classification of Diseases 10; I10) or examined for other conditions for which blood pressure control is important, e.g. cardiovascular disease, previous ischemic stroke or transitory ischemic attack, diabetes mellitus type 2, chronic kidney disease, obesity, hypercholesterolemia, and previously untreated high blood pressure. Exclusion criteria were need for emergency care, advanced stages of disease or dementia, inability to perform self-directed BP measurement, pregnancy, atrial fibrillation, arm circumference too small or wide for the device, lack of consent, or re-entry into the same study. Enrollment was conducted from February 1, 2021, through December 30, 2022.

### Enrollment procedures

**Fig. 1:**
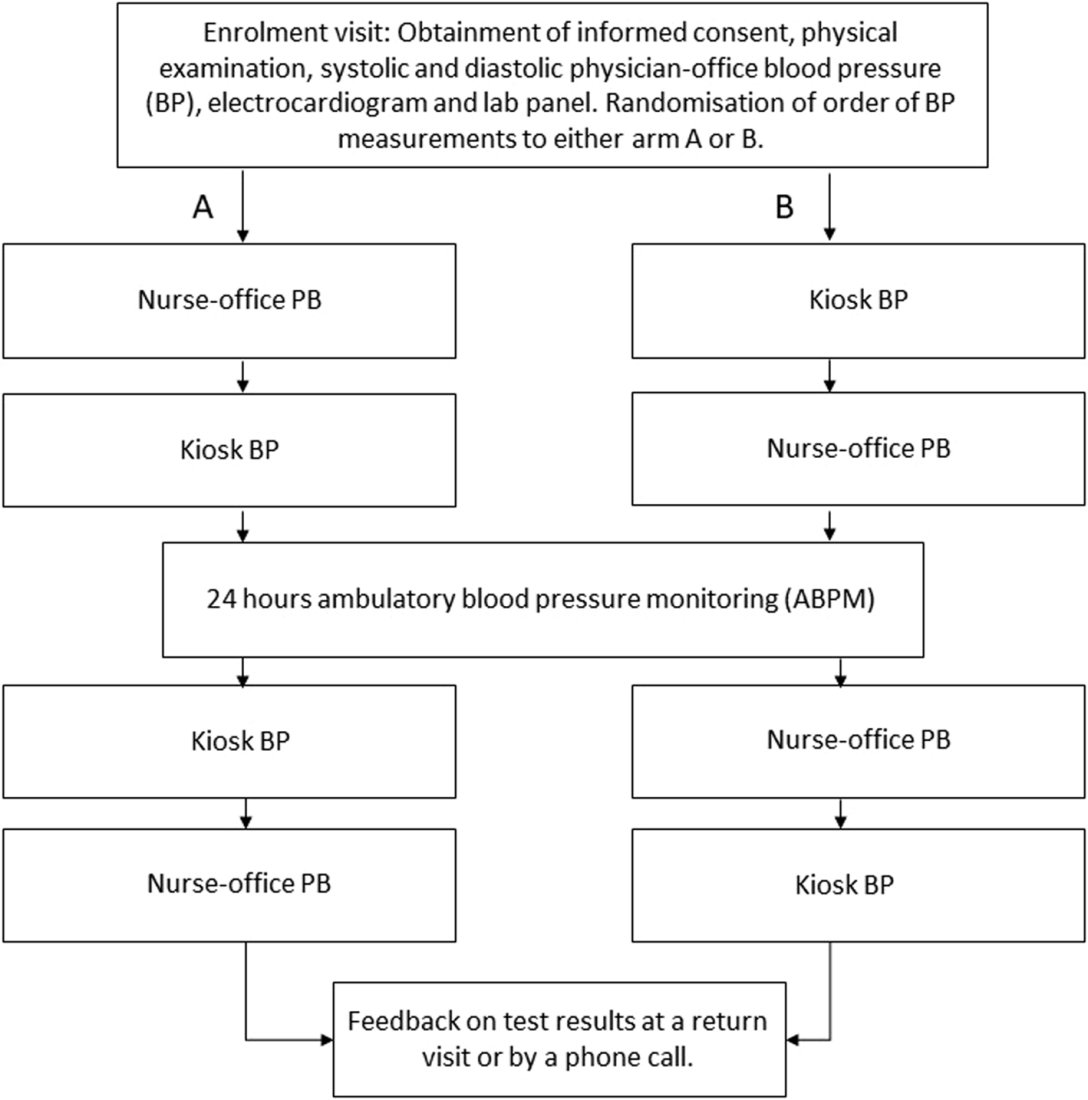
Flowchart of study procedure, Abbreviation BP; blood pressure

All participants underwent a 12-lead electrocardiogram, biochemical panel, physical examination, and physician-measured office BP. The time span from enrollment to ABPM and the last BP measurement in the office or at the kiosk was normally within 2 days, up to a maximum of 7 days, depending on logistics (Fig. 1).

From the enrollment visit through the last study-related BP measurement, study participants stayed on the same medication. The ABPM protocol was available to staff and participants only after the in-office and kiosk BP measurements were completed. Otherwise, there were no blinding procedures.

### Randomization

To randomize participants to kiosk and office BP measurement before ABPM was set up, sealed envelopes were used at the enrollment visit. After participants completed ABPM, the kiosk and office BP measurements were repeated in reverse order (Fig. 1).

### Measurement

BP measurements were performed with the following meters: in-office, Omron M7 Inteli IT (CE. 01997), certified for arm circumferences 22–42 cm; in kiosk, Omron i-Q142 (CE. 0197), certified for arm circumferences 22–42 cm; and for ABPM, Meditech ABPM-05 Blue BP-05 (CE 0120), cuff widths 120–250 mm for 23–33 cm arm circumference and 150–330 mm for 31–40 cm arm circumference. All study equipment was validated by the department of medical technology, Östersund Hospital, before and during the trial on a yearly check-up scheduled.

Kiosk and office BP values were registered after 5 minutes of quiet rest, with the patient sitting in a chair with a backrest, arm supports, and feet on the floor, with the BP cuff on the left upper arm at the level of the heart. Three BP values were recorded at an interval of 1–2 minutes, with further measurements only if the first two values differed by > 10 mmHg. Mean BP was taken using the last two measurements, and the total mean BP was taken as the average of four measurements, two before ABPM in the BP kiosk and two after ABPM, by the nurse in-office. BP at the enrollment visit was recorded as the mean of the last two measurements in a series of three (all physician-measured BP). Trained study nurses conducted the other office BP measures. Kiosk BP was assessed in a separate kiosk for self-directed BP measurement, adjacent to the clinic. Study participants were instructed about using the device before BP measurement in the kiosk, with only one study participant and no staff present in the kiosk. The study participants were instructed to register their BP values by hand on their study forms, and a mean value was subsequently calculated.

ABPM was registered every 30 minutes from 06 A.M. until 22 P.M. and every 60 minutes at night, for a total of 24 hours. The mean value calculated from the measures taken during 06 A.M. to 22 P.M was recorded as the daytime AMBP.

Measurement of in-office BP and ABPM was performed on the left upper arm for comparison with the corresponding measurements in the kiosk, where the device was adapted for BP measurement on the left upper arm, with a button control for each measurement on the right-hand side.

### Outcome measures

The primary outcome was comparative measurements of systolic and diastolic BP among in-office (including nurse- versus physician-measured BP), kiosk, and daytime ABPM values. The secondary outcome was the diagnostic performance of in-kiosk and in-office (including nurse- versus physician-measured) BP, with daytime ABPM as reference. For daytime ABPM and kiosk BP, we defined the diagnostic cut-off as ≥135 mmHg systolic and/or ≥ 85 mmHg diastolic BP. For office BP, we defined the diagnostic cut-off as ≥140 mmHg systolic and/or ≥ 90 mmHg diastolic BP.

### Statistical analysis

In exploratory analyses, we evaluated the performance of ≥140 mmHg systolic and/or ≥ 90 mmHg diastolic BP in kiosk and ≥ 160 systolic and/or ≥ 100 diastolic BP in office and in kiosk, with daytime ABPM as reference. We also evaluated the diagnostic performance of office and kiosk BP measures with the total 24-hour ABPM as reference (diagnostic threshold ≥130 systolic and/or ≥ 80 diastolic for 24-hour ABPM). In addition, we conducted this analysis separately for participants with drug treatment for hypertension. The diagnostic cut-off levels for in-office and kiosk BP and ABPM were selected according to recommended reference standards [4].

For sample size calculations, the difference among BP settings was set at ≥4 mmHg systolic BP with a standard deviation (SD) of 15, nominal power 0.8, alpha 0.05, and an assumed correlation of 0.5. The calculation resulted in a necessary minimum of 113 participants. The estimated SD for systolic BP was based on previous BP registrations in the same population. To account for potential late exclusions and drop-outs, we aimed to recruit up to 150 study participants.

Standard descriptive measures, means, and proportions were presented for baseline variables, and the paired *t*-test (two tailed) was used for comparisons of means. The statistical significance level was set at *p* < 0.05. SPSS (version 25, IBM Corp) and a diagnostic test evaluation calculator (MedCalc Software Ltd.) were used for the analyses [20].

## Results

**Fig. 2:**
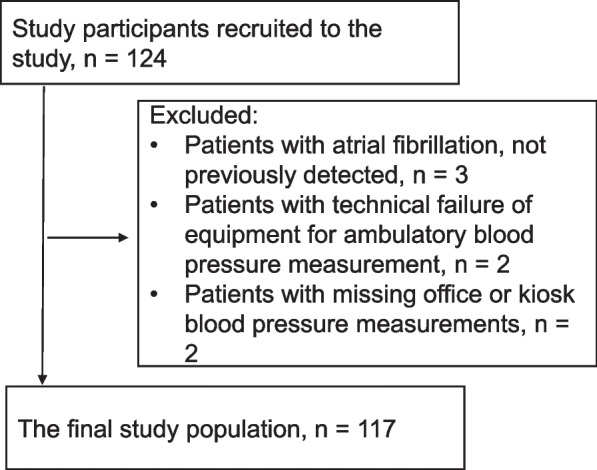
Flowchart of patient inclusion

Of 124 potential study participants, the final study population comprised 117 persons who completed the trial with full data sets (Fig. 2).

The mean participant age was 67 years, and 41% were women. Eighty percent had ongoing treatment for hypertension, and the remaining were visiting for other conditions for which BP monitoring was relevant. For current medication and other participant data, see Table 1.

**Table 1:** Characteristics of study participants (*N* = 117)

**Age, years, mean (SD)**	**67.4 (10.5)**
Female sex, n (%)	48 (41)
Systolic BP, mmHg, mean (SD)^a^	143.6 (15.4)
Diastolic BP, mmHg, mean (SD)^a^	83.3 (11.1)
**Medication at enrollment visit**	**N (%)**
Anti-hypertensive drug treatment	94 (80.3)
Beta-blocker	31 (26.5)
Renin–angiotensin system inhibitor	37 (31.6)
Renin–angiotensin system inhibitor + thiazide diuretic	35 (29.9)
Calcium channel blocker	42 (35.9)
Alpha-blocker	5 (4.3)
Mineral corticoid inhibitor	3 (2.6)
Thiazide diuretic single	8 (6.8)
Loop diuretic single	5 (4.3)
Other anti-hypertensive drugs^b^	9 (7.7)
**Other measures**	
Body mass index, kg/m^2^, mean (SD)	29.4 (5.2)
Estimated glomerular filtration rate, mL/min/1.73 m^2^, mean (SD)	71.9 (11.2)
Uric acid, μmol/L, mean (SD)	327.9 (73.6)

*Abbreviations*: *BP* blood pressure, *HbA1c* glycated hemoglobin

^a^BP, systolic and diastolic = the mean of two measurements at enrollment visit

^b^e.g., combinations of angiotensin receptor blockers and calcium channel blockers

### Primary outcomes

Compared with daytime ABPM, mean kiosk BP was higher by 6.2 (95% confidence interval [CI] 3.8–8.6) mmHg for systolic and 7.9 (95% CI 6.2–9.6) mmHg for diastolic (*p* < 0.001). The mean systolic nurse-measured BP in the office was similar to daytime ABPM values (+ 2.0 mmHg, 95% CI − 0.2–4.2; *p* = 0.071), but the diastolic nurse-measured BP was higher by 7.2 mmHg (95% CI 5.9–9.6; *p* < 0.001). The mean systolic and diastolic physician-office BP measures were higher compared with daytime ABPM values, by 7.6 (95% CI 4.5–10.2) mmHg for systolic and 5.8 (95% CI 4.1–7.6) mmHg for diastolic (*p* < 0.001; Table 2).

**Table 2:** Differences in mean systolic and diastolic BP and daytime ABPM^a^ by type of BP monitoring (*N* = 117)

**Type of BP monitoring**	**Systolic BP (mm Hg)**		***P***
	**Mean (SD)**	**Mean difference in BP daytime ABPM (95% CI)**	
Daytime ABPM (reference)	136.0 (12.9)	NA	NA
Kiosk^b^	142.2 (13.8)	6.2 (3.8–8.6)	< 0.001
Nurse measured ^c^	138.1 (13.2)	2.0 (−0.2–4.2)	0.071
Physician-measured, at enrollment ^d^	143.6 (15.4)	7.6 (4.5–10.2)	< 0.001
**Type of BP monitoring**	**Diastolic BP (mm Hg)**		***P***
	**Mean (SD)**	**Mean difference in BP daytime ABPM (95% CI)**	
Daytime ABPM (reference)	77.4 (9.5)	NA	NA
Kiosk	85.3 (10.2)	7.9 (6.2–9.6)	< 0.001
Nurse-measured	84.6 (8.9)	7.2 (5.9–9.6)	< 0.001
Physician-measured at enrollment	83.3 (11.1)	5.8 (4.1–7.6)	< 0.001

Analyses performed with paired *t*-test, daytime ABPM reference.

^a^Daytime ABPM, mean of ambulatory registrations from 06 A.M. until 22 P.M, every 30 minutes

^b^ Kiosk for self-directed BP monitoring

^c^ Nurse-measured: mean of 4 BP measurements, two before and two after ABPM

^d^Physician-measured at enrollment: mean of 2 BP measurements Abbreviations: *ABPM* ambulatory blood pressure monitoring, *BP* blood pressure, *CI* confidence interval, *SD* standard deviation

Compared with the mean systolic kiosk BP, the systolic nurse-office BP was lower, by − 4.1 mmHg (95% CI − 5.5 to − 2.8; *p* < 0.001), whereas mean diastolic measurements were similar between the kiosk and office values (Table 3). The mean difference between physician-measured and nurse-measured BP was 5.5 (95% CI 3.5–7.6) mmHg for systolic (*p* < 0.01) and − 1.3 (95% CI − 2.8–1.5) mmHg for diastolic (*p* = 0.08).

**Table 3:** Differences between systolic and diastolic BP monitoring performed in office or in a kiosk (*N* = 117)

**Type of BP monitoring**	**Systolic BP (mm Hg)**		***P***
	**Mean (SD)**	**Mean difference in office – kiosk BP (95% CI)**	
Kiosk^a^ (reference)	142.2 (13.8)	NA	NA
Nurse-measured^a^	138.1 (13.2)	−4.1 (−5.5 to −2.8)	< 0.001
Physician-measured at enrolment^b^	143.6 (15.4)	1.4 (−0.8–3.7)	0.215
**Type of BP monitoring**	**Diastolic BP (mm Hg)**		***P***
	**Mean (SD)**	**Mean difference in office – kiosk BP (95% CI)**	
Kiosk (reference)	85.3 (10.2)	NA	NA
Nurse-measured	84.6 (8.9)	−0.7 (−1.9–0.4)	0.205
Physician-measured at enrolment	83.3 (11.1)	−2.1 (−4.0 to −0.2)	0.032

Analyses performed with paired *t*-tests with kiosk BP as reference.

*Abbreviations*: *ABPM* ambulatory blood pressure monitoring, *BP* blood pressure, *CI* confidence interval, *SD* standard deviation

^a^Mean of four measurements in BP kiosk or nurse-measured, 2 BP measurements each day, before and after ABPM

^b^Mean of 2 BP measurements in office by a physician at the enrollment visit

### Secondary outcomes

The diagnostic performance of nurse-measured and kiosk BP monitoring was compared, with daytime ABPM as reference. Nurse-measured BP, with a diagnostic threshold of ≥140 mmHg systolic and/or ≥ 90 mmHg diastolic, had a sensitivity of 66.7% (95% CI 53.7–78.1%), specificity of 72.2% (95% CI 58.4–83.6%), false-positive rate of 12.8%, false-negative rate of 17.9%, and accuracy of 69.2% (95% CI 60.0–77.4%). Kiosk BP monitoring, with a diagnostic threshold of systolic ≥135 mmHg and/or diastolic ≥85 mmHg, had a sensitivity of 88.9% (95% CI 78.4–95.4%), specificity of 38.9% (95% CI 25.9–53.1%), false-positive rate of 28.2%, false-negative rate of 6%, and accuracy of 65.8% (95% CI 56.5–74.3%) (Table 4).

**Table 4:** Diagnostic performance of nurse-measured and kiosk BP values (mm Hg) with daytime ABPM reference ^a^ (*N* = 117)

Daytime ABPM ≥135/85 (reference)	Nurse-measured ≥ 140/90	Kiosk I ≥135/85	Kiosk II ≥140/90
Daytime ABPM positive for hypertension, %	53.8	53.8	53.8
Sensitivity, % (95% CI)	66.7 (53.7–78.1)	88.9 (78.4–95.4)	71.1 (58.7–82.1)
Specificity, % (95% CI)	72.2 (58.4–83.6)	38.9 (25.9–53.1)	66.7 (52.5–78.9)
Positive predictive value, % (95% CI)	73.7 (63.7–81.6)	62.9 (57.4–68.1)	71.4 (62.4–79.0)
Negative predictive value, % (95% CI)	65.0 (55.8–73.3)	75.0 (58.1–86.7)	66.7 (56.5–75.6)
Positive likelihood ratio, (95% CI)	2.40 (1.51–3.82)	1.45 (1.16–1.83)	2.14 (1.42–3.22)
Negative likelihood ratio, (95% CI)	0.46 (0.31–0.68)	0.29 (0.13–0.62)	0.43 (0.28–0.66)
True positive (hypertensive), n (%)	42 (35.9)	56 (47.9)	45 (38.5)
True negative (hypertensive), n (%)	39 (33.3)	21 (17.9)	36 (30.7)
False positive, n (%)	15 (12.8)	33 (28.2)	18 (15.4)
False negative, n (%)	21 (17.9)	7 (6.0)	18 (15.4)
Accuracy, % (95% CI)	69.2 (60.0–77.4)	65.8 (56.5–74.3)	69.2 (60.0–77.4)

*Abbreviations*: *ABPM* ambulatory BP monitoring, *BP* blood pressure, *CI* confidence interval

^a^Diagnostic BP thresholds: ABPM, ≥135 mmHg systolic and/or ≥ 85 mmHg diastolic (reference); nurse-measured, ≥140 mmHg systolic and/or ≥ 90 mmHg diastolic; kiosk, ≥135 mmHg systolic and/or ≥ 85 mmHg diastolic (Kiosk I), or ≥ 140 mmHg systolic and/or ≥ 90 mmHg diastolic (Kiosk II)

At a higher diagnostic threshold of systolic ≥140 mmHg and/or diastolic ≥90 mmHg for the kiosk, the sensitivity decreased to 71.1%, and the specificity increased to 66.7%. The complete characteristics of the diagnostic performances are shown in Table 4. Analysis of data for participants who were on medication for hypertension (*n* = 94) demonstrated only minor differences compared with the complete study cohort (Supplementary Table 1).

Extremely high BP (systolic ≥160 and/or diastolic ≥100) in office or kiosk increased the diagnostic specificity, with only a few false-positive cases for kiosk BP (2/117, 1.7%), for a specificity of 96.3 (95% CI 87.3–99.6), and no false-positive cases with nurse-measured BP. This specificity came at the expense of a lower sensitivity of 19.1 (95% CI 10.3–30.9) in the kiosk and 12.7 (95% CI 5.7–24.0) for nurse-measured values, with daytime ABPM systolic ≥135 and/or diastolic ≥85 as reference.

In the complementary analyses, we noted that the mean kiosk and office BP levels were higher before compared with after ABPM, with approximate differences of 4–6 mmHg systolic and 2–3 mmHg diastolic (*p* < 0.001; (Supplementary Table 2). The diagnostic performance of nurse-measured and kiosk BP values with 24-hour ABPM as reference remained at about the same level as in the primary analyses (Supplementary Table 3).

**Fig. 3:**
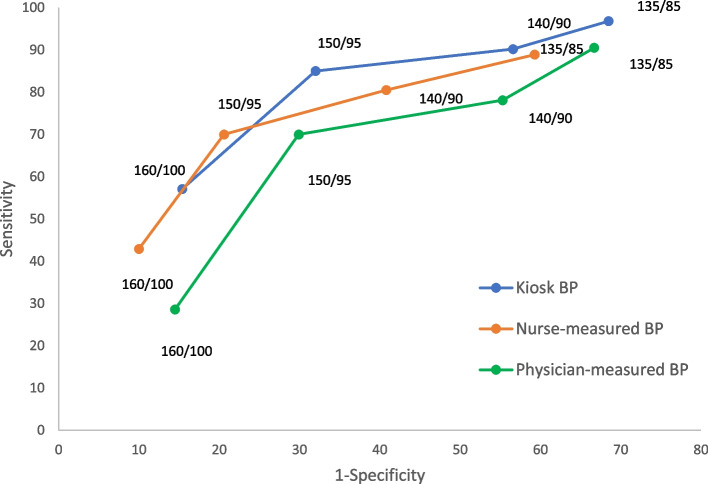
Receiver operating characteristic curves of BP monitoring, in kiosk, nurse-measured, and physician-measured across pairs of systolic/diastolic cut-off levels (daytime ABPM reference)

Receiver operating characteristic curves for BP monitoring in the kiosk and by the nurse or physician in the office across pairs of systolic/diastolic cut-off levels (daytime ABPM as reference) demonstrated overall similar diagnostic performance for kiosk and nurse-measured values, with the curve for physician-measured values landing under (Fig. 3).

## Discussion

This study compared BP monitoring by automated devices in a kiosk for self-determined BP measurement and in the office by nurses and physicians, with daytime ABPM as reference. We also evaluated the diagnostic performance of all three monitoring techniques separately.

Compared with ABPM, mean kiosk systolic BP was about 6 mmHg higher and diastolic BP was 8 mmHg higher. Nurse-measured systolic BP was similar to ABPM, but nurse-measured diastolic BP was higher by 7 mmHg, as was mean physician-measured BP, by about 6–8 mmHg for both systolic and diastolic values. The differences between office and kiosk BP were small, and nurse-measured systolic BP was at most 4 mmHg lower than the mean kiosk BP.

Evaluation of the diagnostic performance of kiosk and office BP revealed only a moderately high accordance with ABPM as reference. Increasing the diagnostic threshold of hypertension from 135/85 to 140/90 in the kiosk decreased the number of false positives, but at the expense of an increased number of false negatives. The moderate diagnostic accuracy of kiosk BP monitoring in our study (65.8%) was below the 77.1% reported in a recent study from 12 Kaiser Permanente primary care centers in Washington state [16]. The diagnostic accuracy of nurse-measured BP monitoring in our setting (69.2, 95% CI 60–77.4%) was similar to that of kiosk BP monitoring (65.8, 95% CI 56.5–74.3%), in contrast to the Kaiser Permanente study, with a reported accuracy of 44.4% for clinic values [16]. The receiver operating characteristic curves based on paired diagnostic thresholds of systolic and diastolic BP indicated a similar diagnostic performance between kiosk and nurse-measured BP and over the curve for physician-measured BP. In the Kaiser Permanente study, the performance of kiosk and home BP monitoring was better than office BP monitoring across a range of systolic and diastolic BP values. In our study at-home BP monitoring was not included. The reason for not including home BP was lack of standardization and experience in our population during the study period.

In the complementary analyses of participants receiving anti-hypertensive treatment (*n* = 94), and in the analysis using 24-hour ABPM as reference, no clear influence on overall diagnostic accuracy was noted, compared with the primary analyses. Extremely high BP readings (≥160/100) in office or in kiosk increased specificity at the expense of lower sensitivity, confirming that white-coat hypertension could be ruled out with some assurance at that level of high BP [16].

Concerning the observed difference in mean systolic and diastolic BP values before and after ABPM, this observation is in contrast to findings by Andreades et al. [21], who could not confirm an order-related effect on automated office BP readings. To avoid order-related bias from nurse-measured and kiosk BP values, in the current study, the order of types of BP monitoring was randomized and repeated in reverse after ABPM. The physician-measured BP was registered only at the enrollment visit. Conclusions related to physician-measured BP compared with the other types of BP monitoring thus should include consideration of this difference.

Circadian variations in BP seems less likely to have influenced our results. Nurse-measured BP and kiosk BP were scheduled during the same visit before set-up of ABPM and repeated in reverse order immediately after having completed ABPM. The enrollment visits were during normal office ours 08.00–17.00 without certain preference for time of the day.

### Limitations

This was a single-center study with a limited sample size, which could have influenced the external validity of our findings. The BP kiosk is not a standardized concept, despite efforts to amend this issue in the last decade. A BP kiosk could be designed and equipped in various ways, all with the common purpose of offering easy access to BP monitoring [22–25]. About four out of every five study participants received treatment for hypertension, and differences between this population and the treatment-naïve participants could not be excluded. The diagnostic performance of all types of BP monitoring depends on the cut-off levels used to define hypertension, an arbitrary parameter that can vary among studies.

Firm conclusions on the external validity of findings in a single-center study should be made with caution. Compared with the population of the QregPV register of region Västra Götaland, Sweden, which allowed recruitment of 259,753 primary care patients with hypertension [26], we recruited a smaller proportion of women (41% vs 56.5%), but mean ages were similar (67.4 vs 68.7 years). In addition, less participants in our study had ongoing drug treatment for hypertension at enrollment (80.3% vs 93.8%).

The advantages of our study approach are that the study equipment was uniformly validated and handled by a limited number of experienced staff, study participants were primary care patients receiving normal care and previously registered at the clinic, and the time span from enrollment to study completion was within 1 week, with no alterations in medication during that period. The BP kiosk was in a quiet room, adjacent to the office. The necessity of standardizing kiosk BP monitoring has been emphasized previously [27].

We believe that our findings can be of use in designing the most appropriate ways to monitor BP and care for the vast number of patients with hypertension [28].

## Conclusions

In this study, kiosk BP monitoring was not comparable to daytime ABPM but could be an alternative to in-office BP monitoring by trained nurses. The diagnostic performance of kiosk and nurse-measured BP was similar and better than physician-measured BP, with daytime ABPM as reference.

### Supplementary Information

**Additional file 1.**

**Additional file 2.**

**Additional file 3.**

## Data Availability

The datasets obtained during the study will be available from the corresponding author on reasonable request.

## Abbreviations

ABPM: Ambulatory blood pressure monitoring
BP: Blood pressure
CI: Confidence interval
SD: Standard deviation
