# Supplementary material for: A comparative trial of blood pressure monitoring in a self-care kiosk, in office, and with ambulatory blood pressure monitoring
Source: BMC Cardiovasc Disord. 2024 Jan 3;24:27. doi: 10.1186/s12872-023-03701-1 (PMC10765747; doi:10.1186/s12872-023-03701-1)
Supplement: Supplementary file 1 — Additional file 1. [file 12872_2023_3701_MOESM1_ESM.docx]

**Supplementary Table 1 Diagnostic performance of nurse-measured and kiosk BP values among patients treated for hypertension, with daytime ABPM as reference* (n = 94)**

| **Daytime ABPM**  **≥135/85 (reference)** | **Nurse-measured BP ≥140/90** | **Kiosk I**  **BP ≥135/85** | **Kiosk II**  **BP ≥140/90** |
| --- | --- | --- | --- |
| Reference positive for hypertension, % | 50.0 | 50.0 | 50.0 |
| Sensitivity, % (95% CI) | 63.8 (48.5–77.3) | 87.2 (74.3–95.2) | 74.4 (59.7–86.1) |
| Specificity, % (95% CI) | 70.2 (55.1–82.7) | 34.0 (20.9–49.3) | 63.8 (48.5–77.3) |
| Positive predictive value, % (95% CI) | 68.1 (56.8–77.8) | 56.9 (51.2–62.5) | 67.3 (57.6–75.2) |
| Negative predictive value, % (95% CI) | 66.0 56.0–74.8) | 72.7 (53.4–86.2) | 71.4 (59.5–81.0) |
| Positive likelihood ratio, (95% CI) | 2.14 (1.31–3.49) | 1.32 (1.05–1.67) | 2.06 (1.36–3.12) |
| Negative likelihood ratio, (95% CI) | 0.52 (0.34–0.79) | 0.37 (0.16–0.87) | 0.40 (0.23–0.68) |
| True positive (hypertensive), n (%) | 30 (31.9) | 41 (43.6) | 35 (37.2) |
| True negative (hypertensive), n (%) | 33 (35.1) | 16 (17.0) | 30 (32.0) |
| False positive, n (%) | 14 (14.9) | 31 (33.0) | 17 (18.1) |
| False negative, n (%) | 17 (18.1) | 6 (6.4) | 12 (12.8) |
| Accuracy, % (95% CI) | 67.0 (56.6–76.4) | 60.6 (50.0–70.6) | 69.2 (58.8–78.3) |

*Diagnostic BP thresholds: daytime ABPM (reference), ≥135 mmHg systolic and/or ≥85 mmHg diastolic; nurse-measured, ≥140 mmHg systolic and/or ≥90 mmHg diastolic; and kiosk, ≥135 mmHg systolic and/or ≥85 mmHg diastolic (Kiosk I) or ≥140 mmHg systolic and/or ≥90 mmHg diastolic (Kiosk II)

Abbreviations: ABPM: ambulatory BP monitoring; BP: blood pressure; CI: confidence interval
