# Supplementary material for: A comparative trial of blood pressure monitoring in a self-care kiosk, in office, and with ambulatory blood pressure monitoring
Source: BMC Cardiovasc Disord. 2024 Jan 3;24:27. doi: 10.1186/s12872-023-03701-1 (PMC10765747; doi:10.1186/s12872-023-03701-1)
Supplement: Supplementary file 2 — Additional file 2. [file 12872_2023_3701_MOESM2_ESM.docx]

**Supplementary Table 2 Mean BP before (I) and after (II) ABPM by type of BP monitoring* (N = 117)**

| **Type of BP monitoring** | **Systolic BP I, mean (SD)** | **Systolic BP II, mean (SD)** | **Mean difference (95% CI)** | **P** |
| --- | --- | --- | --- | --- |
| Kiosk | 144.8 (14.5) | 139.2 (15.9) | 5.6 (3.3–7.9) | <0.001 |
| Nurse-measured** | 140.0 (13.8) | 136.1 (14.5) | 3.8 (2.0–5.7) | <0.001 |
| **Type of BP monitoring** | **Diastolic BP I, mean (SD)** | **Diastolic BP II, mean (SD)** | **Mean difference (95% CI)** | **P** |
| Kiosk | 86.8 (10.9) | 83.7 (11.1) | 3.1 (1.5–4.6) | <0.001 |
| Nurse-measured | 85.7 (9.9) | 83.4 (9.1) | 2.3 (1.0–3.5) | <0.001 |

Analyses performed with paired *t*-tests.

*Mean of two BP measurements, in kiosk for self-directed BP monitoring and nurse-measured, before (I) and after (II) ABPM

Abbreviations: ABPM: ambulatory blood pressure monitoring; BP: blood pressure; CI: confidence interval; SD: standard deviation
