# Supplementary material for: A comparative trial of blood pressure monitoring in a self-care kiosk, in office, and with ambulatory blood pressure monitoring
Source: BMC Cardiovasc Disord. 2024 Jan 3;24:27. doi: 10.1186/s12872-023-03701-1 (PMC10765747; doi:10.1186/s12872-023-03701-1)
Supplement: Supplementary file 3 — Additional file 3. [file 12872_2023_3701_MOESM3_ESM.docx]

**Supplementary Table 3 Diagnostic performance of nurse-measured and kiosk BP values with 24-hour ABPM as reference (N = 117) ***

| **Daytime ABPM**  **≥130/80 (reference)** | **Nurse-measured**  **BP ≥140/90** | **Kiosk BP ≥135/85** |
| --- | --- | --- |
| ABPM positive for hypertension, % | 47.9 | 47.9 |
| Sensitivity, % (95% CI) | 71.4 (57.8–82.7) | 82.3 (78.1–96.0) |
| Specificity, % (95% CI) | 72.1 (59.2–82.9) | 36.1 (24.2–49.4) |
| Positive predictive value, % (95% CI) | 70.2 (60.4–78.5) | 56.2 (51.0–61.3) |
| Negative predictive value, % (95% CI) | 73.3 (63.8–81.0) | 78.6 (61.6–89.3) |
| Positive likelihood ratio, (%) | 2.56 (1.66–3.97) | 1.40 (1.13–1.72) |
| Negative likelihood ratio, (%) | 0.40 (0.25–0.62) | 0.30 (0.13–0.68) |
| True positive (hypertensive), n (%) | 40 (34.2) | 50 (42.7) |
| True negative (hypertensive), n (%) | 44 (37.6) | 22 (18.8) |
| False positive, n (%) | 17 (14.5) | 39 (33.3) |
| False negative, n (%) | 16 (13.7) | 6 (5.1) |
| Accuracy, % (95% CI) | 71.8 (62.7–79.7) | 61.6 (52.1–70.4) |

*Diagnostic thresholds: 24-hour ABPM, ≥130 mmHg systolic and/or ≥80 mmHg diastolic (reference); nurse-measured, ≥140 mmHg systolic and/or ≥90 mmHg diastolic; kiosk, ≥135 mmHg systolic and/or ≥85 mmHg diastolic
Abbreviations: ABPM: ambulatory BP monitoring; BP: blood pressure; CI: confidence interval
